# Roles of sex hormones in mediating the causal effect of vitamin D on osteoporosis: A two-step Mendelian randomization study

**DOI:** 10.3389/fendo.2023.1159241

**Published:** 2023-03-30

**Authors:** Yongwei Du, Baohui Xie, Maoyuan Wang, Yanbiao Zhong, Zhimai Lv, Yun Luo, Qiwei He, Zhen Liu

**Affiliations:** ^1^ Department of Orthopedics, First Affiliated Hospital of Gannan Medical University, Ganzhou, China; ^2^ Department of Orthopedics, Shangyou Hospital of Traditional Chinese Medicine, Ganzhou, China; ^3^ Department of Rehabilitation, First Affiliated Hospital of Gannan Medical University, Ganzhou, China; ^4^ Department of Internal Medicine-Neurology, First Affiliated Hospital of Gannan Medical University, Ganzhou, China; ^5^ Ganzhou Polytechnic, Ganzhou, China

**Keywords:** osteoporosis, vitamin D, sex hormone, Mendelian randomization, causality

## Abstract

**Background:**

Although 25-hydroxyvitamin D [25(OH)D] is a risk factor for osteoporosis, it is not clear whether sex hormones mediate this casual association. We aimed to explore how sex hormones affect the association between 25(OH)D and osteoporosis to provide meaningful insights on the underlying mechanisms from a genetic perspective.

**Methods:**

Genetic variations in 25(OH)D, total testosterone (TT), androstenedione (A4), estradiol (E2), and testosterone/17β-estradiol (T/E2) were determined through summary statistics. Taking osteoporosis as the outcome (FinnGen biobank, 332,020 samples), we conducted a Mendelian randomization (MR) analysis to establish the association between 25(OH)D and these sex hormones. The two-step MR analysis quantified the mediatory effects of sex hormones on osteoporosis. The results were further verified by pleiotropy and heterogeneity analyses.

**Results:**

MR results showed that 25(OH)D (OR= 1.27, *p* = 0.04) and TT (OR= 1.25, *p* = 0.04) had a causal effect on osteoporosis. No significant associations were observed between the other sex hormones (A4, E2, and T/E2) and osteoporosis (*p*>0.05). Sensitivity analysis (*p*>0.05) confirmed the robustness of the MR results. The two-step MR analysis provided evidence that the mediatory effect of TT was 0.014 (the percentage of TT mediation was 5.91%). Moreover, the direct effect of 25(OH)D on osteoporosis was 0.221. A4, E2, and T/E2 were not considered as potential mediators of the role of 25(OH)D as a risk factor for OP.

**Conclusion:**

This study, through MR analysis, showed that TT mediates the causal effect of 25(OH)D on osteoporosis. Interventions targeting TT, therefore, have the potential to substantially reduce the burden of osteoporosis attributable to high 25(OH)D.

## 1 Introduction

Osteoporosis (OP) is currently the most prevalent metabolic bone disorder, characterized by low bone mass and microarchitecture deterioration (1). According to epidemiological estimates, OP affects more than 30% of men and 50% of women (2). In addition, a substantial rise in the prevalence of OP is anticipated due to the increasing aging population, presenting a significant global health burden (3, 4). Therefore, it is meaningful to identify risk factors for OP and explore the in-depth relationship between risk/protective factors.

Vitamin D (VD) affects bone health by regulating calcium absorption and plays a crucial role in OP (5). Notably, 25-hydroxyvitamin D [25(OH)D] can be used as a marker of VD levels, allowing for its effect on OP to be evaluated (6). However, some studies have shown that the intake of VD supplements does not affect the levels of 25(OH)D (7). Additionally, it was reported that VD supplements alone did not give satisfactory results in controlling the risk of OP or fractures (8). Nonetheless, some experts in the field argue that the elderly should continue to supplement for VD (9). Considering the short duration of intervention and follow-up in randomized controlled trials, the long-term effects of 25(OH)D might have been underestimated in these previous studies.

Furthermore, it has been shown that VD exerts its effect on bone health by interacting with sex hormones. For instance, Al-Daghri et al. reported that the level of VD was positively correlated with testosterone (TT) levels (10). Androgens also play a direct and indirect role in bone metabolism. In their research using mouse experiments, Kristine et al. showed that androgens can affect bone resorption (11). Moreover, *in vitro* studies have shown that estrogen can induce osteoclast apoptosis by upregulating the Fas ligand, thus preventing bone loss caused by OP (12). Notably, Srivastava et al. (13) showed that estrogen can reduce the response of osteoclast precursors to RANKL, thus preventing bone loss. However, the metabolism of sex hormones in the human body is a complex and dynamic process, and their effect on bone health is affected by many factors. Therefore, it is difficult to directly determine the relationship between sex hormones and osteoporosis.

Most biases due to acquired confounding factors affect the assessment of OP risk mediated by the interaction of VD and sex hormones. To avoid such effects, Mendelian randomization (MR) can effectively be used to analyze causality from the perspective of genetic variation. Therefore, we performed a two-step MR analysis to examine the contribution of 25(OH)D and sex hormones in the progression of osteoporosis. Additionally, a two-step MR analysis was further used to evaluate the mediatory effect of specific sex hormones. This provides new insights on the prevention and treatment of osteoporosis.

## 2 Methods

### 2.1 Study design

This was a two-step MR study aimed at investigating the effect of 25(OH)D on the risk of osteoporosis. Sex hormones including total TT, androstenedione (A4), estradiol (E2), and testosterone/17β-estradiol (T/E2) were analyzed as mediators. The specific causal analysis included the following steps (1): exploring the effects of 25(OH)D and sex hormones on the risk of osteoporosis (2), assessment of the causal relationship between 25(OH)D and sex hormones, and (3) investigating how sex hormones mediate the role of 25(OH)D as a risk factor for osteoporosis.

### 2.2 Exposure: 25(OH)D GWAS data

25(OH)D data were obtained from a genome-wide association study (GWAS), based on a linear mixed model, consisting of 496,946 UK Biobank (UKB) participants (40–69 years old) (14). The Diasorin Liason^®^ was used for the quantitative determination of 25(OH)D (range, 10–375 nmol L^−1^). After adjusting for the fit age, sex, genotyping batch, and the first 40 ancestry principal components (PCs), 6,896,093 single nucleotide polymorphisms (SNPs) were obtained.

### 2.3 Mediators: Sex hormone GWAS data

Data on TT and E2 were obtained from a GWAS consisting of 194,453 European samples (15). These GWAS data, obtained from “v3” release of the UK Biobank (16) applied the K-means clustering method to the first four principal components. After adjusting for the genotyping chip, age, BMI, and 10 generically derived PCs, 16,131,612 (TT) and 16,136,413 (E2) genetic loci were obtained. The genetic structure in the cohort was explained using the genetic relationship matrix.

Data on A4 and T/E2 were obtained from a GWAS (17) consisting of 3,549 European samples from LIFE-Adult (18) and LIFE-Heart (19). This GWAS used liquid chromatography–tandem mass spectrometry to quantitatively determine A4 levels and employed the electrochemiluminescence immunoassay to quantitatively study both T and E2. After adjusting for age, sex, and log-transformed BMI, 8,783,995 (A4) and 8,822,708 (T/E2) SNPs were obtained.

### 2.4 Outcome: Osteoporosis GWAS data

To explore how sex hormones mediate the role of 25(OH)D as a risk factor for osteoporosis, the FinnGen biobank (sample size: 190,879 women and 151,620 men) (20) was selected as the source of OP GWAS data. A total of 2,202 disease endpoints were included, and 20,175,454 variants were analyzed in FinnGen (Release 8) (20). The GWAS data for OP were obtained from the FinnGen database (Release 8), which included 6,303 OP patients and 325,717 controls.

### 2.5 Quality control of SNPs

In order to obtain reliable instrument variables (IVs), three assumptions for MR were made (1): each IV was associated with 25(OH)D/sex hormone (2); all IVs for 25(OH)D/sex hormones that had passed quality control were not associated with confounders (excluding mediators); and (3) the influence of the IVs for 25(OH)D/sex hormones on the risk of osteoporosis was only mediated by 25(OH)D and sex hormones.

A *p*-value <5×10^−8^ was selected as the threshold for IVs for 25(OH)D, TT, and E2. On the other hand, *p*<1×10^−5^ was selected as the threshold for IVs for A4 and T/E2 due to the relatively few IVs (21). Linkage disequilibrium was set to R^2^ < 0.001 (22), and palindrome SNPs were deleted.

### 2.6 Mendelian randomization and statistical analysis

We used the R software (version 2.22) and package “TwoSampleMR” to complete the MR analysis. The Wald ratio (WR) method is used to evaluate the causal effect of a single IV, while the inverse variance weighted (IVW) approach was mainly employed to evaluate the causal effect of multiple IVs (23). MR-Egger (24) and the weighted median (WM) (25) were used to verify causality. In addition, Odds ratios (ORs) and 95% CI were used to indicate the impact on the risk of osteoporosis. For MR results, *p* < 0.05 was considered a potential association. In addition, mediation analysis was conducted on the mediators (sex hormones) with potential correlation. The effect of 25(OH)D on TT was multiplied by the effect of TT on osteoporosis to obtain the mediatory effect of TT. The causal effect of TT was subtracted from the total effect of 25(OH)D on OP to obtain the direct effect of 25(OH)D on osteoporosis. Moreover, the mediatory effect was divided by the total effect of 25(OH)D on OP to obtain the proportion of mediation by TT. Sensitivity analysis was further used to verify the robustness of the results, and the Cochrane’s Q-test was employed to test for heterogeneity. Finally, the MR Egger intercept and MR-PRESSO (26) were used to test for pleiotropy.

## 3 Results

### 3.1 IVs after quality control

After ruling out SNPs that did not meet the standards (*p*<1× 10^−8^, R^2^<0.001, F>10) for quality control, 45 SNPs were selected as IVs to evaluate the causal relationship between 25(OH)D and OP (**Table 1**). For each sex hormone, 50 (TT), 16 (A4), 11 (E2), and 11 (T/E2) SNPs were selected as IVs to evaluate causality between 25(OH)D and OP (**Table 1**). All IVs were not weak (F>10) and passed the MR Steiger filtering step. More details are presented in **Supplementary Table S1**.

**Table 1:** Selection of IVs after quality control.

Exposure	Samples	IVs	F
25(OH)D	496,946	45	29.77–1455.88
TT	194,453	50	26.17–257.56
A4	3,549	16	19.59–85.15
E2	206,927	11	31.48–319.98
T/E2	5,696	11	19.60–23.04

### 3.2 Effect of 25(OH)D on osteoporosis

**Figure 1:**
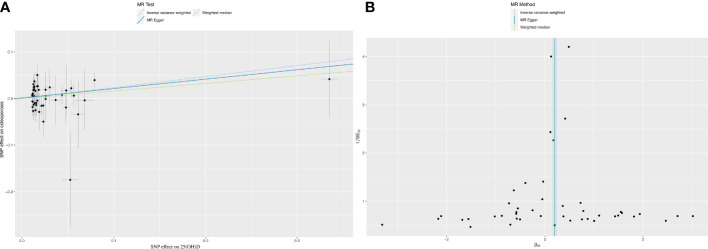
Mendelian randomization analysis between 25(OH)D and osteoporosis. **(A)** The causality between 25 (OH) D and osteoporosis. **(B)** Funnel plot: sensitivity analysis between VD and osteoporosis.

The IVW results revealed a positive association between 25(OH)D and OP (OR=1.27, *p*=0.04) (**Figure 1**). However, the WM (OR=1.18, *p*=0.32) and MR Egger (OR=1.022, *p*=0.24) results showed no association between 25(OH)D and OP (**Figure 1A**). Results from the Cochran’s Q-test confirmed that there was no heterogeneity (IVW, Q=35.73, *p*=0.81; MR Egger, Q=35.65, *p*=0.78). Additionally, the pleiotropy test verified that there was no pleiotropy in the causal relationship between 25(OH)D and OP (intercept=1.40×10^-3^, *p*=0.78, **Figure 1B**). We further used MR-PRESSO to verify the results of pleiotropy (*p*=0.761).

### 3.3 Effect of sex hormones on osteoporosis

**Figure 2:**
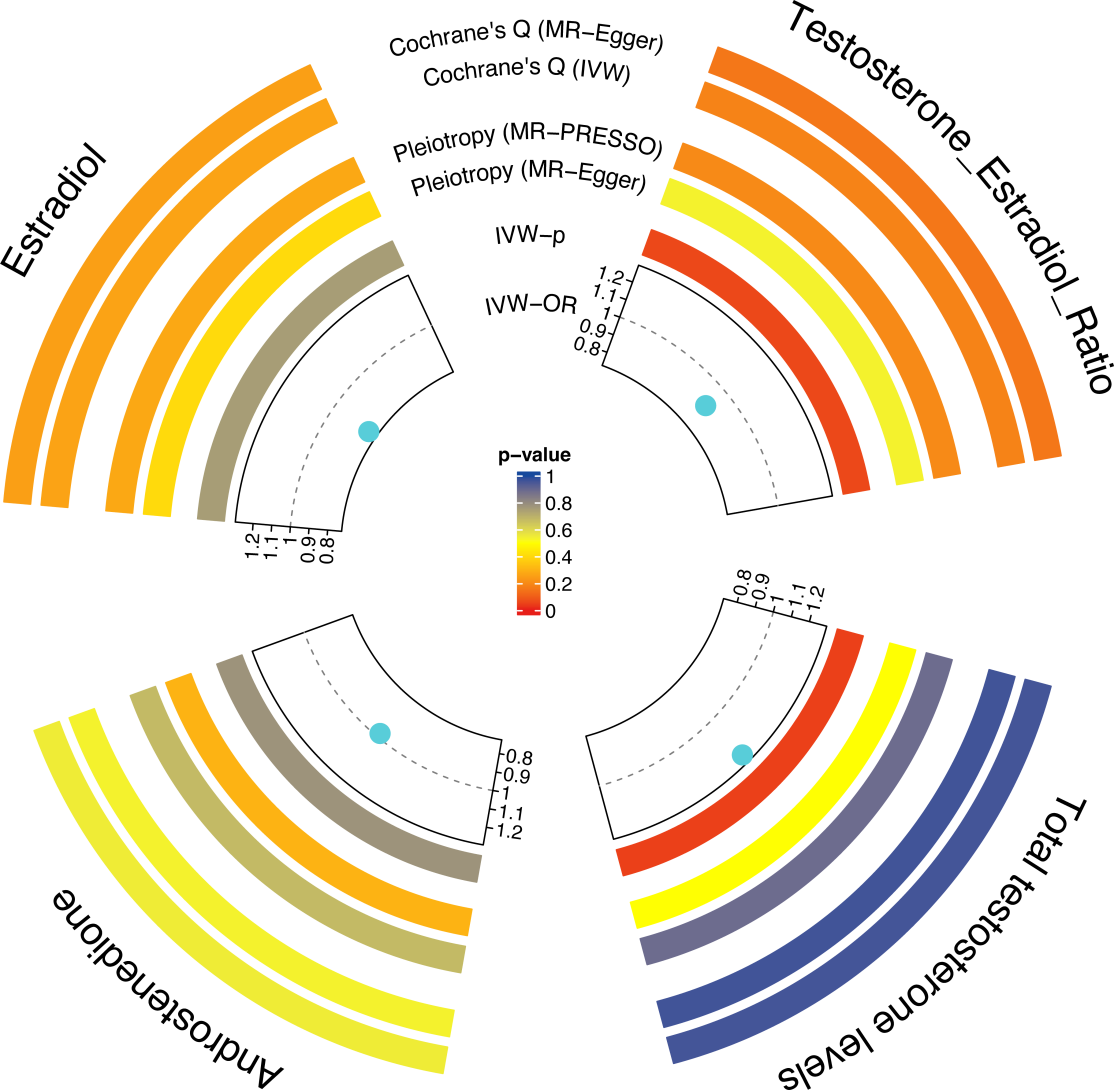
The causal effect of each sex hormone (TT, A4, E2, and T/E2) on osteoporosis. Gray dotted line: OR=1. TT, total testosterone; A4, androstenedione; E2, estradiol; T/E2, testosterone/17β-estradiol.

**Figure 2** shows estimates of the causal effect of each sex hormone on osteoporosis. MR analysis of IVW indicated that TT was associated with a high risk of OP (OR= 1.25; CI, 1.01–1.54; *p*=0.04). However, the causal effect of A4 (OR= 0.98; CI, 0.83–1.15; *p*=0.78), E2 (OR= 0.77; CI, 0.16–3.83; *p*=0.75), and T/E2 (OR= 0.87; CI, 0.76–1.00; *p*=0.05) on osteoporosis was not established. More details of the MR results are shown in the **Supplementary Table S2**.

### 3.4 Mediating effect between 25(OH)D and osteoporosis

Since A4, E2, and T/E2 had no causal effect on osteoporosis, they were not considered as potential mediators of the role of 25(OH)D as a risk factor for OP. Therefore, only TT was subjected to mediation analysis. The findings showed that an increase in TT was positively correlated with the level of 25(OH)D (OR= 1.06; CI, 1.01–1.13; *p*=0.03). Regarding the causal effect of 25(OH)D on osteoporosis, the mediatory effect of TT was 0.014 (the percentage of TT mediation was 5.91%). Moreover, the direct effect of 25(OH)D on osteoporosis was 0.221.

## 4 Discussion

Osteoporosis is one of the most common chronic diseases of the bone that affects older individuals by increasing the risk of fractures, subsequently resulting in a range of complications (27). It is characterized by a substantial reduction in bone quality, caused by deterioration of the bone micro-architecture and low bone mass (28). Moreover, epidemiological data show that more than 200 million individuals worldwide suffer from OP (29). In the aging population, osteoporotic fractures cause significant morbidity and huge cost implications to individuals and the society (30). There are several risk factors for OP, with the most common being age, lifestyle factors, diet, estrogen deficiency, and genetics (31). Furthermore, in both men and women, vitamin D deficiency and sex hormone disorders are the leading causes of OP (32). Given the high prevalence of OP in the elderly, especially post-menopausal OP in elderly women, more research is needed on the causal link between TT, 25(OH)D, and osteoporosis.

Vitamin D is a fat-soluble vitamin that may be obtained through diet and is synthesized endogenously when sunshine stimulates skin production (33). Vitamin D has been linked to bone growth and development and calcium/phosphorus metabolism (34). Vitamin D, in addition to its endocrine role, promotes innate immunity (35). It has been claimed that vitamin D may help to control blood pressure (36). 25-Hydroxyvitamin D is derived from vitamin D and is the final metabolite of vitamin D (37). It is predominantly generated through the hydroxylation of 25-hydroxyvitamin D (25(OH)D) under the supervision of parathyroid hormone (38). Clinically, 25(OH)D is often used to evaluate the intake and utilization of VD. Additionally, 25(OH)D is an essential condition for healthy bones and participates in the secretion and activation of hormones, maintaining the normal level of calcium ions in the blood (39). Vitamin D aids in bone mineralization by facilitating osteoblast derivatization of bone marrow mesenchymal stem cells (BMMSCs). BMMSCs play an essential role in the action of 1,25(OH)2D, which stimulates their differentiation into osteoblasts (40). 25(OH)D induces the differentiation of BMMSCs into osteoblasts at a concentration of 250 nM, and in some cases, this concentration can reach 500 nM (41, 42). According to a previous study, leptin improved the potential of 25(OH)D to stimulate osteoblast differentiation (43). Nonetheless, high levels of serum 25(OH)D3 are considered to be toxic to the body (44, 45). Additionally, there have been several reports on the association between 25(OH)D and biochemical markers of OP (46–48). These studies are consistent with our IVW results, which showed that 25(OH)D had a causal association with osteoporosis. Overall, 25(OH)D is a potential risk factor for OP.

Furthermore, testosterone can act directly on osteoblasts *via* androgen receptors to promote bone formation. It can also have an indirect effect on bone metabolism through its action on different cytokines (49). Notably, the androgen receptor (AR) mediates the action of testosterone on osteoblasts. AR is found in osteoblasts and chondrocytes and can therefore induce bone formation (50). Moreover, testosterone promotes cell differentiation and apoptosis in osteoblasts and chondrocytes (50), and the aromatase enzyme converts TT to estradiol, which prevents osteoporosis (51). Testosterone deficiency further leads to activation of the nuclear factor kappa-B ligand (RANKL) from osteoblasts, although this also helps in promoting osteoclast differentiation (52). Additionally, a number of observational surveys have investigated the link between TT and OP and found a positive correlation between the two (50, 53–55). Here, through MR analysis of IVW, we observed that TT was associated with a high risk of OP.

Compared to observational studies, MR analyses are less susceptible to reverse causality or other potential confounding effects. Therefore, MR analysis may be more beneficial in determining causality. Human Leydig cells express the CYP2R1 gene and vitamin D receptor (VDR) (56). Intriguingly, patients with testiculopathies have lower testicular CYP2R1 gene expression, and 25(OH)D levels significantly decreased in patients after bilateral orchiectomy (56). Both VD and TT increase sperm motility and activity by not only inducing the survival of human spermatozoa but also inducing estrogen secretion from osteoblasts, thus synergistically promoting bone formation. Moreover, D’Andrea et al. (57) reported a positive relationship between total testosterone levels and 25(OH)D, suggesting that hyperandrogenemia can prevent future incidence of osteoporosis, similar to our findings. We subjected TT to mediation analysis and observed that an increase in the levels of TT was positively correlated with the levels of 25(OH)D. Additionally, the causal effect of 25(OH)D on OP was partly mediated by TT.

Although more reliable conclusions can be drawn using the large-scale genetic variation data presented herein, some limitations of the study still need to be considered. First, since all genetic variants were from European populations, the generalization of our findings to other settings needs further validation. Second, MR analysis of T/E2 and OP showed possible causality (*p*=0.05), which may have been due to the small sample size in the T/E2 GWAS study. In future studies, we aim to further explore the intrinsic link between VD, sex hormones, and OP based on individual-level data to provide more theoretical evidence for the prevention and treatment of osteoporosis.

In conclusion, our study provides insights on the role and effects of VD on OP, mediated by sex hormones, and broadens the understanding of the link between VD and osteoporosis. From a clinical perspective, the prevention and control of OP may need to focus on the regulation of sex hormones (especially TT).

## Data availability statement

The original contributions presented in the study are included in the article/**Supplementary Material**. Further inquiries can be directed to the corresponding author.

## Ethics statement

The data involved in this study are from public summary data. The ethical approval of each study can be found in the original publications. All studies followed the ethical guidelines of the Declaration of Helsinki.

## Author contributions

YD and ZLi designed the study, analyzed the data, and drew the figures. All authors critically revised the manuscript. All authors contributed to the article and approved the submitted version.
